# Functional Properties, Rheological Characteristics, Simulated Digestion, and Fermentation by Human Fecal Microbiota of Polysaccharide from *Morchella importuna*

**DOI:** 10.3390/foods13132148

**Published:** 2024-07-06

**Authors:** Shurong Wang, Dongjie Li, Guangle Li, Naixin Duan, Chang He, Junlong Meng, Yanfen Cheng, Xueran Geng, Ludan Hou, Mingchang Chang, Lijing Xu

**Affiliations:** 1 College of Food Science and Engineering, Shanxi Agricultural University, Taigu 030801, China; wzlj2005@163.com (S.W.); ldj19992021@163.com (D.L.);; 2 Shanxi Engineering Research Center of Edible Fungi, Taigu 030801, China

**Keywords:** *Morchella importuna polysaccharide*, processing properties, simulated fermentation, gut microbiota, hypoglycemic

## Abstract

*Morchella importuna* polysaccharide (MIP) has been proven to have obvious hypoglycemic effects on mice with type 2 diabetes (T2DM). This study looked at the functional and rheological characteristics of MIP, and investigated the effects of MIP on the human fecal microbiota through in vitro fermentation experiments. The outcomes demonstrate the excellent oil-holding capacity, emulsifying, foaming, and rheological characteristics of MIP. After salivary gastrointestinal digestion, the Mw of MIP decreased from 398.2 kDa and 21.5 kDa to 21.9 kDa and 11.7 kDa. By 16S rRNA sequencing of bacteria fermented in vitro, it was found that MIP did not improve the richness and diversity of intestinal microorganisms, but it may exert an anti-T2DM function by significantly increasing the relative abundance of Firmicutes and promoting *Ruminococcaceae_UCG_014*, *Bacteroides*, and *Blautia* proliferation. *Escherichia-Shigella* could also be inhibited to improve the intestinal microenvironment. In addition, the fermentation of MIP increased the total short-chain fatty acid (SCFA) concentration from 3.23 mmol/L to 39.12 mmol/L, and the propionic acid content increased significantly. In summary, MIP has excellent processing performance and is expected to exert potential anti-T2DM activity through the human intestinal microbiota, which has broad market prospects.

## 1. Introduction

Numerous studies have demonstrated that natural polysaccharides are beneficial for glycolipid metabolism, anti-inflammatory activity, anti-tumor activity, and reducing oxidative stress, and these biological activities are associated with their prebiotic features [1]. Due to polysaccharides being unable to be absolutely digestible by the stomach, it is essential to produce large amounts of SCFAs through the degradation and utilization of host intestinal microorganisms to reshape the composition of intestinal microorganisms and to improve the intestinal environment [2]. Therefore, polysaccharides as prebiotics have been widely concerned and utilized, such as inulin [3], Arabic gum [4], Chinese yam polysaccharide [5], and *Dendrobium candidum* polysaccharide [6]. *Morchella esculenta*, a precious edible and medicinal mushroom worldwide, has been utilized in traditional medicine to cure digestive disorders, sputum production, and shortness of breath in China for centuries [7]. With the maturity of the technology, its rich nutrients and excellent therapeutic properties are gradually known, among which the bioactivities of *Morchella* polysaccharides have been extensively studied, including immune regulation [8], antioxidation [9], nervous system protection [10], and cholesterol-lowering activity [11], which indicates that it has broad potential to be developed as food, medicine, and healthcare products.

To apply natural polysaccharides to food or biomedicine, it is necessary to study their processing characteristics. It is well known that polysaccharide processing is mainly related to functional properties and rheological characteristics [12,13]. The emulsifying and foaming properties and water and oil retention of polysaccharides directly affect the texture and quality of processed foods [14]. The rheological properties of food products may be altered with the addition of polysaccharides in food formulations. The functional and rheological feature of other polysaccharides can only provide a limited reference because of different materials in the rough sources and extraction processes [15]. Therefore, studying the functional properties and rheological characteristic of *Morchella importuna* polysaccharide (MIP) is necessary.

The metabolic condition known as diabetes mellitus (DM) is typified by elevated blood glucose levels, which not only produce insulin resistance but also bring a series of serious complications. According to the estimation of the International Diabetes Federation, there were over 463 million diabetics globally in 2019; by 2030, there will be 578 million, and by 2045, there will be 700 million [16]. Over 90% of diabetes is T2DM, which is mainly caused by unhealthy eating habits, obesity, mental stress, and heredity, and often requires lifelong medication [17]. Retaining blood glucose at a suitable level is a crucial strategy for managing the diabetes risk. α-Glucosidase inhibitors are applied for the initial cure of diabetes or in combination with other drugs to treat diabetes. This inhibition reduces glucose absorption and achieves the effect of lowering blood glucose levels after meals; while α-glucosidase inhibitors, including acarbose, voglibose, and miglitol, are frequently utilized to lower blood glucose levels after meals, they are associated with side effects such as bloating and abdominal pain [18]. It is worth noting that natural polysaccharides have significant hypoglycemic activity without adverse side effects, and have become an important source of antidiabetic drugs and health food with hypoglycemic function. The *M. importuna* polysaccharides extracted with deep eutectic solvents [19], *Evodiae fructus* polysaccharides [20], and *Ampelopsis grossedentata* polysaccharide [21] have favorable inhibitory activity on α-glucosidase. Meanwhile, numerous papers have now demonstrated an apparent correlation between T2DM and gut microorganisms. Polysaccharides may improve the relative abundance (RA) of favorable gut bacteria, increase the yield of SCFAs, and increase the profitable liver metabolites via the gut–liver axis, thereby reducing the incidence of T2DM [22,23,24]. Large metagenome-wide studies have documented gut microbial dysbiosis as regarding T2DM [25]. The disorder of the gut microbiota will destroy the intestinal barrier, affect glucose metabolism and lipid metabolism, and then lead to insulin resistance and T2DM, which suggests that the intestinal microbiota is probably a novel drug target in the treatment of T2DM [23]. In vitro fermentation can clarify the impact of polysaccharides upon human intestinal microorganisms by studying compositions of the intestinal microbiota and changes in its metabolites [26], which can not only be used to screen and develop different prebiotics and probiotics but also be used to study the mechanism of polysaccharide fermentation and transformations of xenobiotic substances and generate hypotheses [27]. At present, it has not been reported whether MIP can be effectively decomposed and utilized by human intestinal microorganisms or which microorganisms play a major role in this process. Therefore, we studied the in vitro fermentation of MIP and expounded on the changes in the fermentation fluid, microbial population, and SCFAs during the fermentation process so as to conduct a preliminary study on the regulatory effect of MIP on intestinal microorganisms.

Recently, we obtained crude MIP from *M. importuna*, and preliminarily studied its monosaccharide composition, infrared spectrum, particle size and potential, thermal stability, and in vitro hypoglycemic and hyperlipidemic activity, and determined its glucoside linkage by a methylation analysis [28]. In our previous study, MIP ameliorated glucose intolerance in T2DM mice via the gut–liver axis, in which the gut microbiota played an important role [22]. However, the digestive physiology and intestinal microbial composition of animals and humans are not exactly the same, and modeling digestive tracts in vitro represents a powerful tool for studying the microbial composition of the gut [27]. Therefore, we hope to make a thorough inquiry of the influence of MIP on the intestinal microbiota through in vitro fermentation and to lay a theoretical basis for developing and applying MIP to hypoglycemic health food by studying the processing characteristics of MIP.

## 2. Materials and Methods

### 2.1. Materials and Reagents

*M. importuna* fruit bodies were provided by Shanxi Engineering Research Center for Edible Mushrooms (Jinzhong, Shanxi Province, China). MD44 dialysis membranes (molecular weight cutoff, 3500 Da), α-amylase from *Bacillus subtilis* (3700 U/g), bovine serum albumin (BSA), free cholesterol assay kit, and pepsin (1:10,000) were provided by Solarbio Science & Technology Co., Ltd. (Beijing, China). Pancreatin, bile salts, and carboxymethyl cellulose were acquired from Sigma-Aldrich (St. Louis, MO, USA). Peptone, yeast extract, vitamin K1, hemin, Tween-80, and resazurin were purchased from Yuan Ye (Shanghai, China). All of the other reagents and chemicals were analytically pure.

### 2.2. MIP Preparation

*M. importuna* powder and distilled water were mixed at a ratio of 1:30, extracted at 80 °C for 2 h, and the supernatant was combined after repeated extraction 3 times. After protein removal by potassium ferrocyanide and zinc acetate, then dialysis and precipitation by alcohol, MIP was obtained after freeze-drying.

### 2.3. Functional Properties

#### 2.3.1. Water- and Oil-Holding Capacities

The water-holding capacity (WHC) and oil-holding capacity (OHC) were measured through use of a method provided by Jeddou et al. [29]. Regarding the WHC, dry MIP (1 g) was put into a tube and weighed, to which 20 mL deionized water was added, and the mixture was left for 30 min. After being centrifuged at 8000× *g* for 10 min, the supernatant was removed and the residue was weighed again.

The following formula was used to calculate the WHC:

(1) $$WHC(g/g)=\frac{Water\;absorbed\;weight(g)}{Sample\;weight(g)}$$

Regarding the OHC, MIP (0.5 g) was added into the tube and weighed; then, 10 mL soybean oil was added, following by standing for 30 min. After being centrifuged at 8000× *g* for 10 min, the supernatant was removed and the residue was weighed again.

The following formula was used to calculate the OHC:

(2) $$OHC(g/g)=\frac{Oil\;absorbed\;weight(g)}{Sample\;weight(g)}$$

#### 2.3.2. Emulsion Properties

The method of Wang et al. [30] was utilized to measure the emulsion stability (ES) and emulsion capacity (EC) of MIP. MIP was dissolved in distilled water to different concentrations (0.5, 1, 2, 4, and 6%; *w*/*v*). After mixing with soybean oil at a ratio of 1:1 (*v*/*v*), the solution was homogenized (FLUKO, Shanghai, China) at 10,000 rpm for 2 min.

The following formula was used to calculate the EC:

(3) $$EC(\%)=\frac{Emulsion\;volume}{Total\;volume}\times 100$$

For ES, the emulsion was bathed in water at 80 °C for 30 min, followed by cooling to room temperature and then centrifugation (1000× *g*, 10 min).

The following formula was used to compute the ES:

(4) $$ES(\%)=\frac{Final\;emulsion\;volume}{Total\;volume}\times 100$$

#### 2.3.3. Foaming Property

The method proposed by Wang [30] was utilized to explore the foaming stability (FS) and foaming capacity (FC). MIP solutions (0.5, 1, 2, 4, and 6%; *w*/*v*) underwent homogenization at 10,000 rpm for 2 min. The total suspension volume, the volume after homogenization for 2 min, and the volume after 30 min were measured.

The formulas for computing FC and FS are as follows:

(5) $$FC\left(\%\right)=\frac{Initial\;foam\;volume}{Total\;suspension\;volume}\times 100$$

(6) $$FS\left(\%\right)=\frac{Final\;foam\;volume}{Total\;suspension\;volume}\times 100$$

### 2.4. Rheological Behavior Measurements

In conformity with the method proposed by Xu et al. [31], a conical CP 50-1 plate (50 mm in diameter, 1.0 mm gap) was adopted to conduct steady-state shear and oscillation measurements on an MCR 102 rheometer (Anton-Paar, Graz, Austria).

#### 2.4.1. Steady Shear Flow Behavior

The steady-state shear range was set to 0.1 to 1000 s^−1^, and the influences of various MIP concentrations (1, 2, 3, 4, 5, 6, 7, 8 and 9%, *w*/*v*), pH (1, 3, 5, 7, 9 and 11), and Na^+^ or Ca^2+^ (0.01, 0.3, 0.5, 0.7, 0.9 and 1 mol/L) upon the apparent viscosity (AV) of MIP solutions were measured at 25 °C. When studying temperature factors, the conditions were 10, 20, 30, 40, 50, and 60 °C (all factors except the concentration were tested with a 5% MIP solution).

#### 2.4.2. Oscillatory Shear Measurements

The MIP solutions were prepared with different mass fractions (5%, 7%, and 9%, *w*/*v*), different pH values (3, 7, and 11), and different salt ion concentrations (0.3 mol/L and 0.5 mol/L Na^+^ or Ca^2+^). Setting the temperature to 25 °C, the frequency scanning mode was adopted and the storage modulus (G′), loss modulus (G″), and loss angle (tanδ = G″/G′) of MIP solutions in the range of angular frequency (0.1~100 rad/s) were obtained under a fixed oscillatory strain of 6%.

### 2.5. In Vitro Simulation of Digestion Assay

#### 2.5.1. In Vitro Simulated Saliva–Gastrointestinal Digestion of MIP

Saliva electrolyte solution (SSF), small intestine electrolyte solution (SIF), and stomach electrolyte solution (SGF) were made by referring to previous papers [32,33]. The simulated saliva consisted of 40 mL of SSF and 0.81 g of α-amylase, and the simulated gastric juice consisted of 1.0 M CH_3_COONa (pH 5.0, 0.4 mL), 40 mL of SGF, and 0.12 g of pepsin. The simulated small intestinal fluid comprised 3.0 mL of trypsin (10%, *w*/*v*), 3.0 mL of SIF, and 6.0 mL of the bile acid salt solution (4%, *w*/*v*).

The simulated saliva–gastrointestinal digestion was studied in vitro using a previous method with slight adjustments [34]. In the simulated oral stage, after blending the MIP solution (4 mg/mL) with simulated saliva at 1:1 (*v*/*v*), the mixture was adjusted to a pH value of 7 and vibrated on a shaking table at 37 °C for 3 min. In the stomach stage, after mixing the salivary digestion solution with simulated gastric juice at 1:1 (*v*/*v*), the mixture was adjusted to a pH value of 3 and vibrated on a shaking table at 37 °C for 2 h. In the small intestine stage, the salivary gastric digestion fluid was mixed with simulated small intestinal juice at 10:3 (*v*/*v*), adjusted to a pH value of 7, and vibrated on a shaking table at 37 °C for 2 h. The three liquids after digestion in saliva, stomach, and intestine were inactivated by boiling water for 10 min. After centrifugation, the supernatant was dialyzed, precipitated with anhydrous ethanol, and freeze-dried. The digestion products were separately labeled as the salivary digestion sample (MIP-S), gastric digestion sample (MIP-G), and small intestinal digestion sample (MIP-I).

#### 2.5.2. Molecular Weight Distribution of MIP and Its Digestion Products

The molecular weights of MIP, MIP-S, MIP-G, and MIP-I were measured by high-performance gel permeation chromatography (HPGPC). Prepare the sample into a 5 mg/mL solution, centrifuge it at 12,000 rpm for 10 min, filter the supernatant, and transfer it to the injection bottle. Use a BRT105-104-102 gel column (8 × 300 mm) in series to analyze the sample, use a 0.05 M NaCl solution as mobile phase, and set the flow rate at 0.6 mL/min [35].

### 2.6. In Vitro Fermentation Assay

#### 2.6.1. In Vitro Fermentation of MIP

The fermentation method of Chen et al. [36] was modified slightly. Fresh stool samples were obtained from four healthy adult volunteers (half male and half female) with no gastrointestinal tract infection history and probiotic or antibiotic treatments within three months. Informed consent was obtained from all human subjects, and the same amount of feces from various volunteers was diluted and blended with phosphate buffer (10%, *w*/*v*). After centrifuging the fecal suspension (265 g, 5 min), the filtrate was retained as a bacterial suspension after filtration. After the basic nutrient medium was manufactured in conformity with the method of Guo et al. [37], the pH was adjusted to 7 and sterilization was performed. In the fermentation test, a suspension of the original bacteria, medium without a carbon source, inulin-containing medium, and MIP-containing medium were used as negative control (OR), blank control (BLK), positive control (INL), and experimental group (MIP), respectively. After being added to 9 mL of basal medium, 1.0 mL of 10% fecal pulp was fermented for 48 h using the Anaero Pack system at 37 °C. The INL group and MIP group were treated with 100 mg of inulin and 100 mg of MIP, respectively. Some of the samples were removed at 0, 6, 12, 24 and 48 h for subsequent analysis.

The sample collection process involved in this study did not pose any physical, psychological, legal, or informational risk to the volunteers. In our study, the fecal flora was only used to ferment polysaccharides, and the research content did not involve biomedical research directly related to human health. This study was launched in December 2021, and the reference standard is the “Measures for the Ethical Review of Biomedical Research Involving Human Beings” implemented by the Central People’s Government of the People’s Republic of China on 1 December 2016. This standard does not explicitly specify that the intestinal microbiota excreted by the human body is a human biological sample.

#### 2.6.2. Analysis of In Vitro Fermented MIP Products

##### Determination of OD_600_, Total Carbohydrates, C_R_, pH, and Uronic Acid

The OD_600_ of the fermentation liquid and the pH value were separately determined by an Agilent UV9100 A spectrophotometer and a pH meter (STARA211, Thermo, Bremen, Germany). The C_R_, total carbohydrate, and uronic acid contents of the MIP hydrolytic products were measured based on previous methods [5,36,37].

##### Analysis of the Gut Microbiota

Forty-eight hours after fermentation, an EZNA DNA kit (Omega Bio-Tek, Norcross, GA, USA) was used to collect the total genomic DNA from each sample (*n* = 3). Ouyi Biotechnology Co., Ltd. (Shanghai, China) was entrusted to perform V4 variable region 16S ribosomal RNA (16S rRNA) gene sequencing. After data preprocessing, QIIME2 and VSEARCH software were used to generate cluster operation taxa for clean reads and screen different species [38].

##### Determination of Volatile SCFAs

After filtering the fermentation broth (1.8 mL) from each stage using a 0.22-μm filter membrane, 3% (*w*/*w*) phosphoric acid buffer (0.15 mL) prepared in advance was added. After mixing and filtering using a 0.22-μm filter into a 2.5 mL chromatographic bottle, the SCFA content was determined via gas chromatography (TRACE™ 1300E, Thermo, Germany), with some modifications, by referring to the method proposed by Zhou et al. [39]. The HP-5MS capillary columns (300 mm × 0.25 mm × 0.25 μm) were set to 100 °C for 1 min at first, followed by heating at 4 °C/min to 180 °C for 4 min. Both the detector and injector were maintained at 250 °C.

### 2.7. Data Analysis

Statistical analysis using R language and MS-Excel^®^ 2021 was employed to draw the table in this paper. Data were expressed in the form of means ± standard deviations (SDs). Using SPSS 20.0 software to perform one-way analysis of variance, the data were subjected to multiple comparison tests (Duncan tests) and an analysis of the significance of differences. *p* < 0.05 suggested statistical significance.

## 3. Results and Discussion

### 3.1. Functional Properties of MIP

#### 3.1.1. WHC and OHC

**Figure 1:**
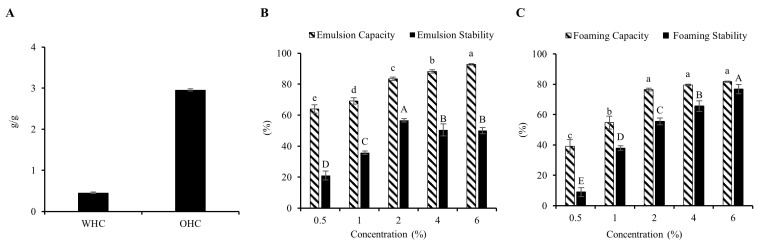
WHC and OHC (**A**), emulsion properties (**B**) and foaming properties (**C**) of MIP. Univariate analysis of variance and Duncan’s test were used for different concentrations, and different uppercase and lowercase letters represent significant differences between different groups (*p* < 0.05); 120 × 117 mm (300 × 300 DPI).

To evaluate functional properties of food systems, it is essential to analyze the WHC and OHC of polysaccharides. The results are shown in Figure 1A. The MIP had a WHC value of 0.91 ± 0.03 g water/g. This is more approximated to formerly published polysaccharides obtained from *R. roxburghii* Tratt fruit (0.25 ± 0.04 g water/g) [30], tamarind seed mucilage (0.18–1.07 g water/g) [13], and *T. foenum-graecum* seeds (11.5%) [14]. Polysaccharides with a strong capacity to bind oil are able to decrease fat uptake in the bloodstream [40]. The OHC of MIP was determined as 5.92 ± 0.04 g oil/g, which was greater compared with those of polysaccharides from chickpea flour [41] and potato peels [29] (3.15 g oil/g and 4.398 ± 0.04 g oil/g). The results revealed that MIP possesses an excellent OHC and holds the potential to improve food properties, such as taste, aroma, texture, and nutrition.

#### 3.1.2. Emulsion Properties

Emulsifiers can increase the quality and taste of food and enhance the stability of products that are crucial in food processing [18]. The emulsifying properties of MIP were evaluated by the indices EC and ES. With increasing MIP concentration, the EC values gradually increased and reached 92.8% at a concentration of 6% (Figure 1B). Moreover, the EC value of MIP was 83.4% at a concentration of 2%, which was significantly greater as compared to polysaccharides extracted from *Rosa roxburghii* Tratt fruit (40.1%) and β-D-glucan from button mushrooms (64.26%) [30,42]. When the concentration was 2%, the ES value was the highest (56.6%). This was more stable than that of okra polysaccharides (38.6%) [43]. The results demonstrated that MIP exhibited considerably good emulsifying properties.

#### 3.1.3. Foaming Properties

The FS and FC of MIP are indicated in Figure 1C. MIP exhibited a concentration-dependent increase in foaming properties. It could produce a rich foam at a low concentration, and these foams were relatively stable. This behavior might be due to MIP improving the viscosity of aqueous phases and forming networks that stabilize interfacial films [44]. In addition, polysaccharides from different sources exhibit different foaming properties. Compared to MIP, the polysaccharides obtained from *Trigonella foenum-graecum* seeds, *R. roxburghii* Tratt fruit, and button mushroom form less foam and show lower stability [14,30,42]. These findings pointed to the good potential for MIP in the food industry.

### 3.2. Steady Shear Flow Behaviors

#### 3.2.1. Influence of the Concentration on the MIP AV

**Figure 2:**
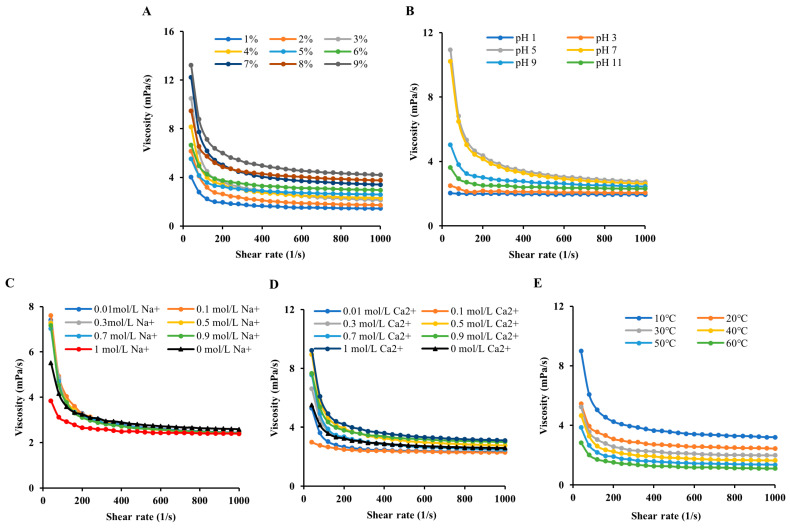
Effects of concentration (**A**), pH (**B**), NaCl (**C**), CaCl_2_ (**D**) and temperature (**E**) on the AV of MIP; 150 × 100 mm (300 × 300 DPI).

How the shear rate influences the AV of MIP has been examined. The results (Figure 2A) were indicative of an elevated AV with rising concentration as a result of the decreasing distance between polysaccharide molecules caused by the high polysaccharide content, which formed more entangled aggregates and caused higher flow resistance [45]. The AV was gradually reduced to a stable level with increased shear rate at different concentrations. This shear-thinning behavior suggests a typical non-Newtonian fluidity, which is primarily due to the long and randomly positioned polysaccharide chains hooking each other at a low shear rate. When in the opposite situation, the process encouraged the polysaccharide chains to gradually arrange in the direction of flow and led to fewer interactions between adjacent polysaccharide chains [46]. This shear-thinning behavior provides the capability to apply MIP to food processing on account that it is easy for liquid food to be mixed and pumped.

#### 3.2.2. Influence of pH on the MIP AV

How the variation of pH from 1 to 12 influences AV is indicated in Figure 2B. The variation in pH can significantly change the MIP AV. MIP showed higher AV in a slightly acidic–neutral pH range (pH 5–7). Beyond that range, the AV decreased. In particular, the viscosity decreased sharply under acidic conditions (pH 1–3). This phenomenon might be because acidic or alkaline conditions lead to hydrogen bond cleavage, polysaccharide decomposition, and a decreased molecular weight, ultimately leading to reduced viscosity [47]. The dependency on pH was similar to that of polysaccharides from *Amana edulis* [48].

#### 3.2.3. Influence of Metal Ions on the MIP AV

Metal ions exerted a degree of impact on the MIP AV. From Figure 2C, its observed that AV grew as the concentration of sodium ions was augmented and declined at high concentrations (1 mol/L sodium ions). This might be because an increase in the amount of NaCl added reduced the MIP molecular charge and encouraged the formation of soluble polysaccharide molecular complexes, resulting in an increment of AV. At higher concentrations of sodium ions, the salting-out phenomenon was more widespread, which resulted in polysaccharide flocculation and precipitation and a reduction in AV [48].

The addition of calcium chloride to the MIP solution led to a decrease in AV, which may be because the electrostatic repulsion between polysaccharides was shielded [49]. The AV improved at a CaCl_2_ concentration higher than 0.3 mol/L (Figure 2D). The increase in the AV may result from an increased number of intermolecular associations [50]. Such a phenomenon was not in agreement with the polysaccharide from *Hericium erinaceus* [51], which may be associated with differences in mushroom polysaccharide components.

#### 3.2.4. Influence of Temperature on the MIP AV

Temperature influences the conformation and gel characteristics of macromolecules by impacting intramolecular and intermolecular hydrogen bonding strengths and the motion speed of molecular chains, and so it has been widely studied as an indispensable influencing factor the rheological characteristics of polysaccharides [52]. The temperature-dependent AV of MIP was researched (Figure 2E). The 5% MIP exhibited decreased AV as the temperature rose from 10 °C to 60 °C. These results demonstrated that a raise in temperature brought about a viscosity decrease under a constant shear rate. This was mainly because of the intensification of molecular motion at high temperatures, which contributed to the larger intermolecular distance and the weaker interaction between the polysaccharide molecules. Similar behavior has also been observed for polysaccharides extracted from puka gum [53] and exopolysaccharide (EPS-BMS) [54].

### 3.3. Oscillatory Shear Measurements

**Figure 3:**
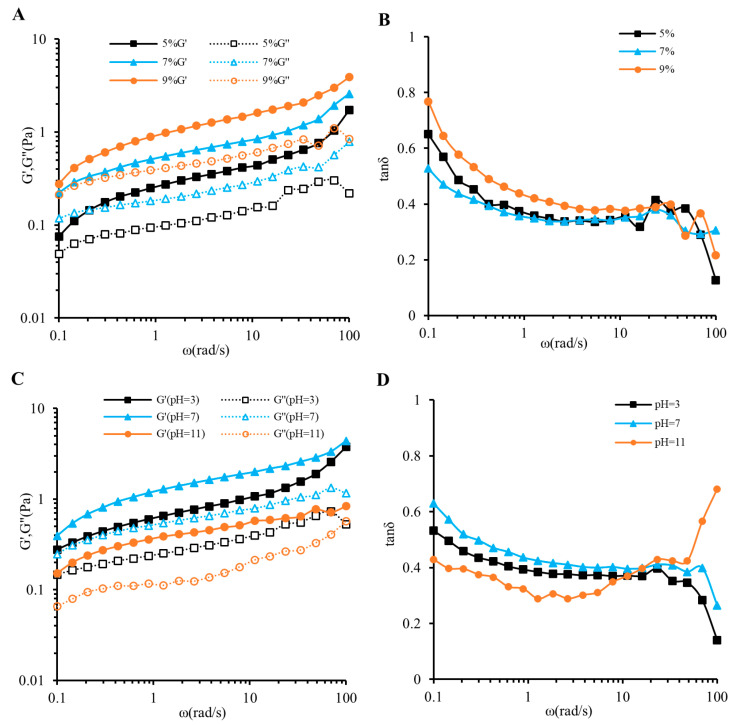
Effects of concentration on the MIP (**A**) storage (G′) and loss (G″) moduli and (**B**) loss tangent (tanδ); effect of pH on the MIP (**C**) storage (G′) and loss (G″) moduli and (**D**) loss tangent (tanδ); 120 × 118 mm (300 × 300 DPI).

**Figure 4:**
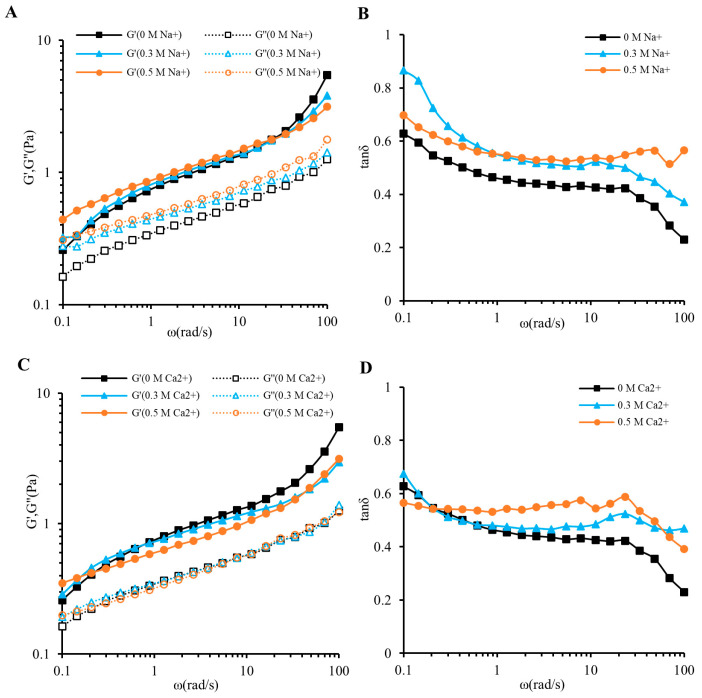
Effects of NaCl on the MIP (**A**) storage (G′) and loss (G2033) moduli and (**B**) loss tangent (tanδ); effects of CaCl_2_ on the MIP (**C**) storage (G′) and loss (G″) moduli and (**D**) loss tangent (tanδ); 120 × 118 mm (300 × 300 DPI).

Viscoelastic measurements can bespeak the gel property of the measured object and are widely used in colloid production. Oscillatory shear measurements can ascertain the property (elasticity or viscidity) of the fluid [55]. G′ and G″ are separately the energy storage and loss moduli. The loss tangent (tanδ = G″/G′) reflects the relative strength of the viscous component and elastic component in the sample. When tanδ < 1, it indicates that the elasticity of the sample is dominant. When tanδ > 1, the viscosity is dominant. A tanδ value higher than 0.1 means that the sample is not a real gel and its structure is between a high-concentration polymer and a real gel [56,57]. The figures show the effects of concentration, pH (Figure 3), Na^+^, and Ca^2+^ (Figure 4) on the storage modulus G′, loss modulus G″ and tanδ of MIP solutions. Within test ranges, G′ was invariably higher than G″ and the tanδ value (0.12–0.76) was lower than 1 but higher than 0.1, which was a typical weak gel behavior. This manifested entanglement between chains was only temporary, and high shear rates could destroy the connections between molecules, manifesting as shear thinning behavior in steady-state experiments (Figure 2) [57,58].

As shown in Figure 3A, the G′ and G″ of MIP solutions were also augmented with rising concentrations, and showed a frequency dependence within the measurement range. The tanδ curve trends were similar for different concentrations, and tan was always less than 1 (Figure 3B), indicating that the concentration of polysaccharides that can form a weak gel was 5–9%. Under alkaline and acidic conditions, G′ and G″ values decreased, but G′ was always greater than G″, which indicated that acidic or alkaline conditions did not destroy the gel structure of the polysaccharide solution (Figure 3C). The tanδ value showed a relatively stable decrease at lower shear rates, which may represent the enhancement of the gel structure. However, when the shear rate reached 100 rad/s, the tan values were alkaline from large to small (0.68), compared with neutral (0.26) and acidic (0.14), indicating that the polysaccharide gel network was not stable under alkaline conditions, which may be due to reduced intermolecular association or shielding effect (Figure 3D) [54]. In addition, adding NaCl to the MIP solution increased the loss modulus (G″) (Figure 4A), and adding CaCl_2_ decreased the storage modulus (G′) (Figure 4C). This is shown in Figure 4B,D as a decrease in tanδ, indicating that adding salt ions decreased the viscoelasticity of the polysaccharide solution, but the polysaccharide solution still maintained a gel state, and similar conclusions were also found for the polysaccharides extracted from *Ocimum album* L. seed [59]. In summary, the MIP solution has good viscoelasticity, and a strong acidic condition, concentration and metal ion addition have little effect on its gel properties, which indicates that MIP is expected to become an excellent substitute for some industrial food thickeners.

### 3.4. Analysis of the Molecular Weights of MIP and Its Digestion Products

The average molecular weights of MIP are 398.2 kDa and 21.5 kDa in Table 1. The molecular weight of MIP decreased gradually during simulated digestion in vitro. After digestion by gastric juice, the molecular weight decreased to 19.1 kDa and 11.6 kDa. After intestinal digestion, the Mw distribution of MIP-I was 21.9 kDa and 11.7 kDa. Compared with MIP-G, there was no significant change, indicating that small intestinal digestion did not affect the molecular weight of MIP, which is consistent with litchi polysaccharide in vitro [60]. The above results indicate that MIP can enter large intestine via the digestive system, and so in vitro fermentation of MIP should be more deeply studied.

### 3.5. In Vitro Fermentation of MIP by the Human Gut Microbiota

#### 3.5.1. Variations in OD_600_, Total Carbohydrate, C_R_, pH, and Uronic Acid Contents after In Vitro Fermentation

**Figure 5:**
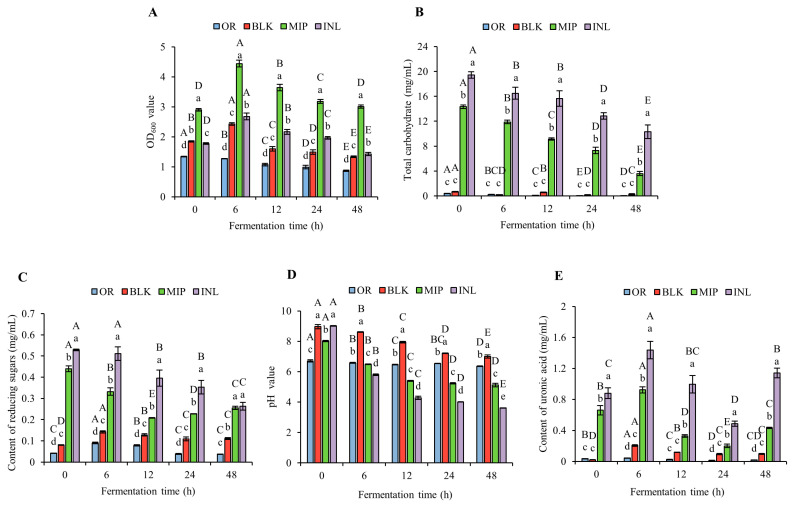
Changes in the in vitro fermentation characteristics of MIP. (**A**) OD_600_; (**B**) total carbohydrate content; (**C**) content of reducing sugars; (**D**) pH value; and (**E**) content of uronic acids. Data are expressed as the means ± standard deviations (*n* = 3), and different lowercase letters represent statistical significance (*p* < 0.05). OR, negative control group (suspension of bacteria); BLK, blank group (no additional carbon sources); MIP, experimental group (MIP supplement); INL, positive control group (inulin supplement). Different capital letters indicate that the same sample has statistically significant differences at different times, and different lowercase letters indicate that there are statistical differences between different samples at the same time point; 150 × 100 mm (300 × 300 DPI).

Figure 5 displays variations in OD_600_, total carbohydrate, C_R_, pH, and uronic acid contents in the in vitro fermentation process. On account of the light brown color of MIP solutions, the OD_600_ values of the MIP group were greater than those of the other groups at 0 h fermentation, and a similar situation occurred in the fermentation of litchi polysaccharides [60]. According to Figure 5A, the continuous decline of the OD_600_ value in the OR group may be due to the gradual decrease in the microbial population due to the lack of a carbon source. The OD_600_ value of the other groups increased at first and then decreased during the whole fermentation, and was always larger when compared with that of the OR group. It is worth noting that in different periods, the OD_600_ values of the MIP group were substantially greater when compared with those of the INL group. The content of residual carbohydrate in MIP fermentation broth was only 25.1% of the beginning value, while the C_R_ content in INL fermentation broth accounted for 53.0% of the beginning value (Figure 5B), indicative of a higher contribution of MIP to the proliferation of intestinal microbiota.

Enzymes from the human intestinal microbiota are able to break down many indigestible polysaccharides. After breaking the glycosidic bond, reducing sugars are produced, which are used for the growth and metabolism of intestinal microorganisms [61]. Figure 5C shows that the C_R_ content of the INL group decreased continuously, and the same conclusions were procured in the fermentation assay of *Crassostrea gigas* polysaccharide in vitro [62], but the content of C_R_ in the MIP group reduced first and then increased. It can be inferred that the gut microbiota can fully use C_R_ released by INL and MIP within 0–12 h. However, the slower increase in C_R_ in the MIP fermentation broth probably due to the higher degradation rate of intestinal microorganisms, which could not completely consume the released C_R_. A comparable tendency was also found in uronic acid, but the recovery appeared at 48 h, which was later than that of C_R_ (24 h) (Figure 5E). This is probably because uronic acid is more easily used by gut microbes during in vitro fecal fermentation compared with other free monosaccharides [63].

pH is an invaluable criterion of the polysaccharide fermentation process. In Figure 5D, the pH of the OR group did not alter conspicuously during 0–48 h, while that of the other groups showed a stable decreasing trend (*p* < 0.05). The least reduction was found in the BLK group (from pH 8.96 to 7, ΔpH = 1.96), the INL group dropped the most (from pH 9.01 to 3.61, ΔpH = 5.4), and the MIP group decreased from pH 8.01 to 5.12, ΔpH = 2.89. Studies have shown that the compositions of microbial colonies are significantly different during in vitro fermentation with a pH of 6.5 and 5.5. Therefore, it can be speculated that the decrease in pH induced by the supplementation of MIP probably influences the compositions of bacterial colonies and the metabolites of gut microbes [64]. The reduced pH of the fermentation broth was mainly because SCFAs were generated during the process, but may also be connected to the increase in the uronic acid content.

#### 3.5.2. Influences of MIP on the Gut Microbiota

**Figure 6:**
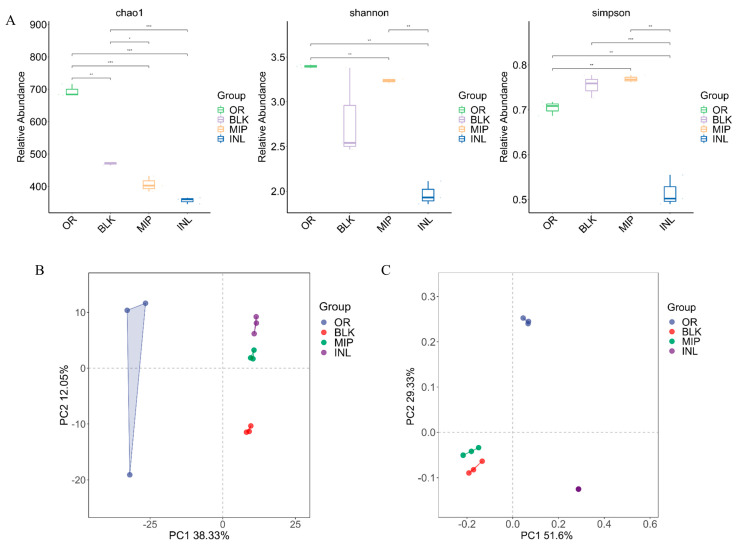
α Diversity (**A**), principal component analysis (**B**), and principal coordinate analysis (**C**) of microorganisms fermented by MIP in vitro. OR, negative control group (suspension of bacteria); BLK, blank group (no additional carbon sources); MIP, experimental group (MIP supplement); INL, positive control group (inulin supplement); 145 × 100 mm (300 × 300 DPI). The difference between the two groups was statistically significant are marked (*) for (*p* ≤ 0.05), marked (**) for (*p* ≤ 0.01) and marked (***) for (*p* ≤ 0.001).

The 16S rRNA sequencing technology has been utilized to represent bacteria after in vitro fermentation. The α and β diversities of each treatment group are shown in Figure 6. The Chao1 index is a community abundance index, and the Shannon index and Simpson index can characterize microbial diversity. The data showed that there were no significant differences in the Chao1, Shannon and Simpson indices between the MIP group and BLK group, indicating that MIP had no obvious effect on improving the richness and diversity of the intestinal microbiota. Principal coordinate analysis (PCoA) and principal component analysis (PCA) were adopted to reflect the effects of different treatment groups on microorganisms. The results showed that the MIP, INL and BLK groups had good separation effects and the samples gathered together in the same group, which showed that MIP could interfere and affect intestinal microorganisms, and so it was necessary to carry out a follow-up analysis.

**Figure 7:**
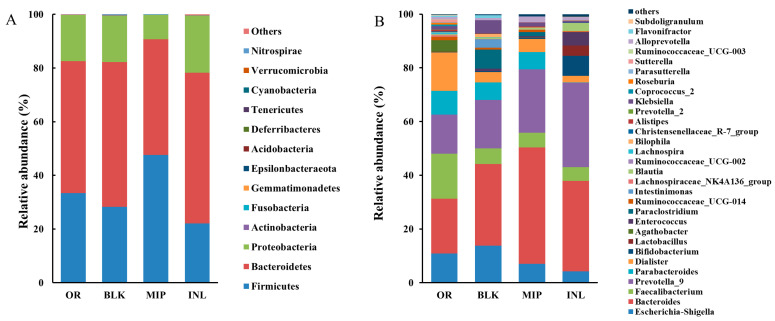
Effect of MIP on the RA of the intestinal bacterial community at the (**A**) phylum level and (**B**) genus level. OR, negative control group (suspension of bacteria); BLK, blank group (no additional carbon sources); MIP, experimental group (MIP supplement); INL, positive control group (inulin supplement); 150 × 60 mm (300 × 300 DPI).

Figure 7A illustrates the bacterial classification and composition distribution at the phylum level of samples. When the 48h fermentation ended, the dominating bacterial groups were Firmicutes, Bacteroidetes, and Proteobacteria, but there were great differences in their proportions. The RA of Firmicutes exhibited a significant increase in the MIP group, while those of Bacteroidetes and Proteobacteria decreased. Our previous study found that the intragastric administration of MIP in T2DM mice also increased the Firmicutes abundance and decreased the Bacteroidetes abundance [22]. Tight junctions (TJs) and mucins are critical for preserving the integrity of the gut barrier. Studies have revealed that hyperglycemia can induce intestinal leakage by altering TJs and adhesion junctions, causing exogenous substances to enter the circulatory system and damage pancreatic beta cells, which is an essential pathway in the pathogenesis of diabetes [65]. Firmicutes can promote the assembly of TJs and the synthesis of mucin, while Bacteroidetes plays the opposite role. In addition, the increase in pathogenic bacteria represents a critical characteristic of gut microbiota in diabetic patients. Many pathogenic bacteria in Proteobacteria, including *Shigella*, *E. coli*, *Helicobacter*, *Escherichia*, *Campylobacter*, and *Salmonella*, are deemed to be connected with high expression of enteric proinflammatory factors [66,67]. The changes in abundance at the phylum level of the gut microflora suggest that MIP may have potential advantages in preventing diabetes.

Figure 7B shows the genus-level distribution of the gut microbiota in various groups. The picture exhibited that *Escherichia-Shigella*, *Faecalibacterium, Bacteroides*, and *Prevotella_9* were the principal bacteria in these groups. The amount of *Bacteroides* in the MIP group was substantially increased. Studies have shown that *Bacteroides* basically take part in the breakdown of carbohydrates such as pectin and have positive effects on sugar metabolism and energy absorption [68]. In addition to a study showing a marked decrease in *Bacteroides* abundance in T2DM patients with progressed glucose intolerance, an analysis of the intestinal microbiota in patients treated with antidiabetic drugs also indicated that abundance of *Bacteroides* grew substantially [69,70]. *Escherichia-Shigella* is significantly connected to various diseases, such as Cushing’s syndrome, tuberculous meningitis, and IgA nephropathy. A significant augmentation of *Escherichia-Shigella* was noted in all of these sufferers and the enrichment degree of *Escherichia-Shigella* in T2DM patients was also improved compared with that in healthy people [71,72,73,74]. Compared with the BLK group, the MIP group showed a remarkable decrease in the number of *Escherichia-Shigella*, proving that MIP can reduce pathogenic bacterial proliferation in the gut and provide a healthier environment for the intestinal system.

**Figure 8:**
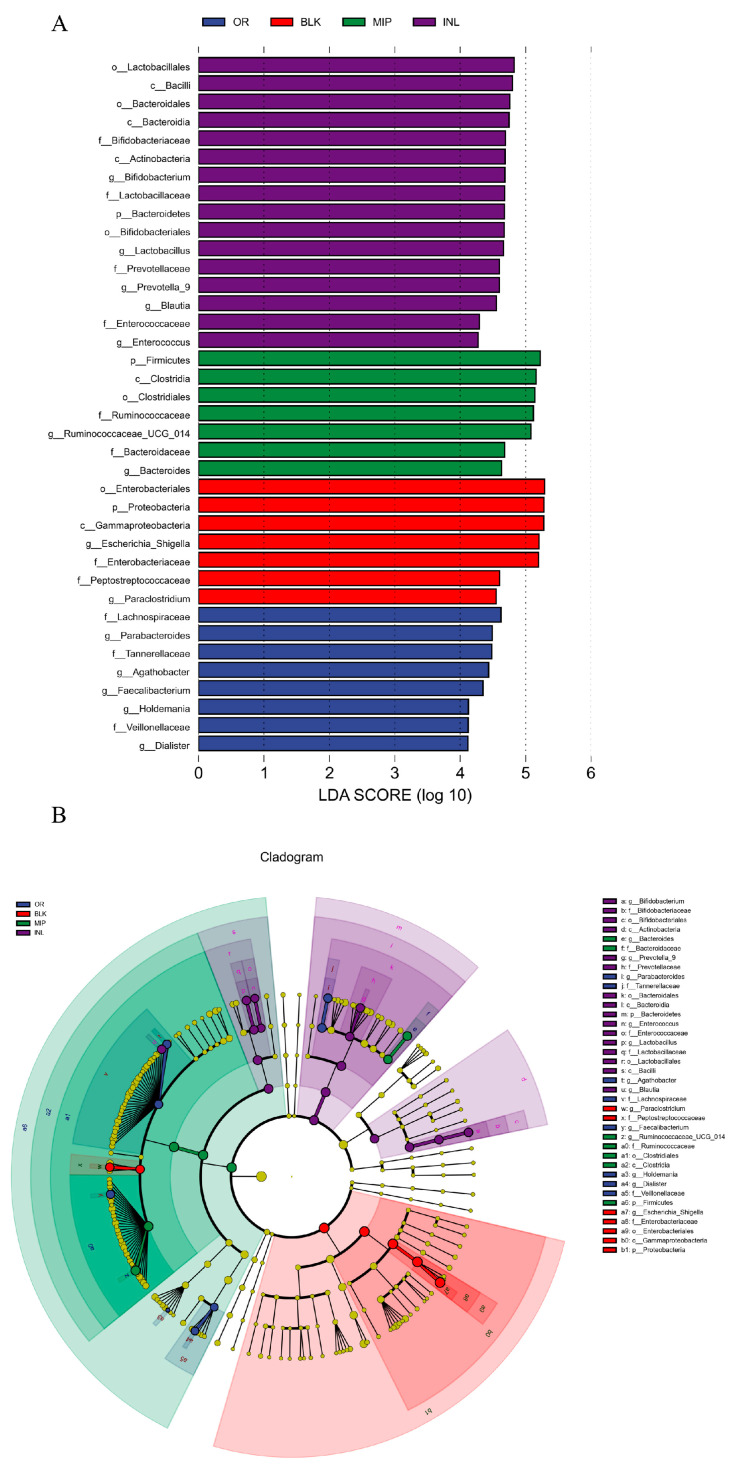
Histogram of the LDA value distribution (**A**) and evolutionary branching diagram (**B**) of microorganisms between different groups. OR, negative control group (suspension of bacteria); BLK, blank group (no additional carbon sources); MIP, experimental group (MIP supplement); INL, positive control group (inulin supplement); 160 × 90 mm (300 × 300 DPI).

The LEfSe analysis includes an LDA value distribution histogram (Figure 8A) and evolutionary branching diagram (Figure 8B). The results showed a total of 38 potential biomarkers of the intestinal microbiota in each group (an LDA score > 4 indicated a statistical difference). At the genus level, the dominant bacterial were *Bifidobacterium*, *Lactobacillus*, *Prevotella_9*, *Blautia,* and *Enterococcus* in the INL group, and *Ruminococcaceae_UCG_014* and *Bacteroides* in the MIP group. *Blautia* can promote SCFA production, and previous research has indicated that oral administration of *Blautia* can alleviate lipid accumulation and hyperglycemia in T2DM mice [75]. Our previous study found the capability of MIP in increasing the RA of *Blautia* in the gut of T2DM mice [22]. In vitro fermentation results also showed that MIP could slightly promote the proliferation of *Blautia*, but the effect of inulin was more significant. In addition, the new findings suggest that MIP could significantly increase the RA of *Ruminococcaceae_UCG_014*, and its decreased RA has been observed in T2DM mice [76]. In summary, although MIP has little effect on the diversity and abundance of intestinal microorganisms, it can improve the intestinal environment by inhibiting pathogenic bacteria (*Escherichia-Shigella*) and enriching probiotics (Bacteroides, *Blautia*, and *Ruminococcaceae_UCG_014*), and may have potential anti-T2DM biological activity.

#### 3.5.3. Changes in the SCFA Content in the Fermentation Broth

Intestinal microorganisms can secrete a variety of sugar-active enzymes, such as sugar esterase, glycoside hydrolase, glycosyltransferase, and polysaccharide lyase. Therefore, polysaccharides are degraded by intestinal microorganisms as they pass through the intestines, and the main product is SCFAs [77,78]. SCFAs can improve T2DM in many ways, including the promotion of insulin secretion, improvement of insulin sensitivity, activation of intestinal gluconeogenesis, and increment of energy consumption [79].

**Figure 9:**
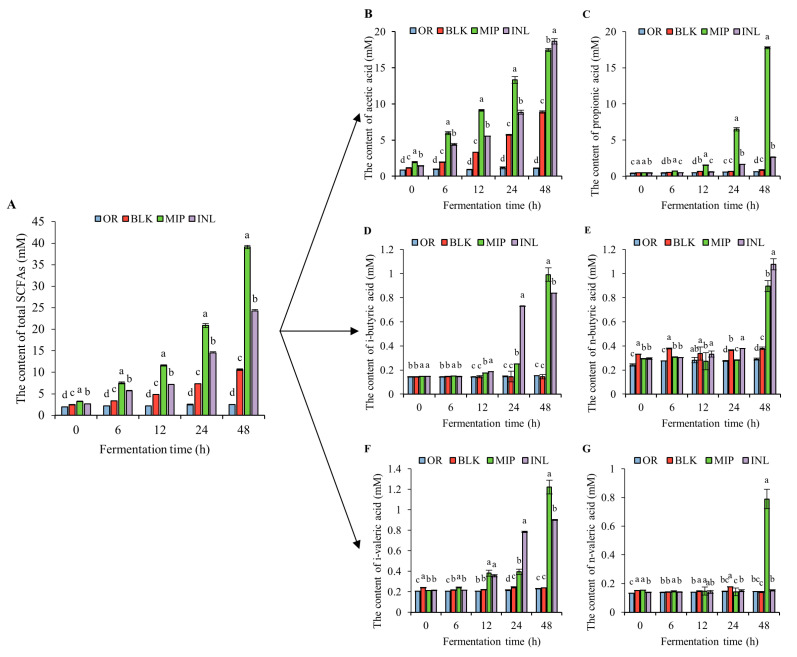
Changes in the contents and total amounts of short-chain fatty acids in fermentative products. (**A**) Total SCFAs; (**B**) acetic acid; (**C**) propionic acid; (**D**) i-butyric acid; (**E**) n-butyric acid; (**F**) i-valeric acid; and (**G**) n-valeric acid. OR, negative control group (suspension of bacteria); BLK, blank group (no additional carbon sources); MIP, experimental group (MIP supplement); INL, positive control group (inulin supplement). Different lowercase letters indicate that there are statistical differences between different samples at the same time point (*p* < 0.05); 150 × 110 mm (300 × 300 DPI).

The SCFA levels in the fermentation broth at various time points during fermentation were determined via gas chromatography (Figure 9). The SCFA yield in all groups showed an increasing trend during fermentation. The MIP group exhibited an increase in SCFAs from 3.23 mmol/L to 39.12 mmol/L, which was noticeably larger when compared with the INL group (from 2.68 mmol/L to 24.28 mmol/L). The main reason is that the MIP group produces more propionic acid than the INL group, which may be because MIP improves the RA of Firmicutes and makes Firmicutes produce more propionic acid through the lactic acid pathway [80]. In vivo and in vitro research has shown that propionic acid is capable of increasing glucose-stimulated insulin release and maintaining healthy glucose homeostasis by protecting islet cells and maintaining beta cell quality [81]. Meanwhile, the acetate-to-propionate ratio in the MIP group decreased from 3.96 (0 h) to 0.98 (48 h) during fermentation, and the low ratio was considered to have a better inhibitory effect on cholesterol synthesis in the liver. MIP may reduce the amount of fat stored by changing the ratio between the two, which has potential beneficial effects on T2DM patients showing disordered glucose and lipid metabolism [82]. It is notable that, compared with the above results, despite its effects on increasing the contents of propionic, acetic, and butyric acids in the stool of T2DM mice, MIP had a weak effect upon the increase in the propionic acid content [22]. In conclusion, MIP can be decomposed and used by gut microorganisms for the production of a diversity of SCFAs, which have good prebiotic potential for resisting T2DM.

## 4. Conclusions

In this study, we found that MIP has good processing characteristics. The WHC value is 0.91 ± 0.03 g water/g and the OHC value is 5.92 ± 0.04 g oil/g. Rheological measurements show that MIP has good viscoelasticity over an extensive pH scope and metal ion addition. The molecular weight of MIP is significantly altered by in vitro digestion. The 16S rRNA sequencing results show that adding MIP increases the growth of some beneficial bacteria such as *Ruminococcaceae_UCG_014*, *Bacteroides*, and *Blautia* and reduces that of harmful bacteria, including *Escherichia-Shigella*. During the in vitro fecal fermentation process, MIP is fully utilized by intestinal microorganisms, which increases SCFs production, and the propionic acid content increases significantly compared to the other groups. The increase in the SCFA concentration and the change in the gut microbiota suggest that MIP may have great potential to prevent T2DM. Therefore, this study provides a theoretical basis for developing and utilizing MIP in functional food and the pharmaceutical industry. However, because there was no in vitro fermentation of the feces of T2DM patients, the intervention effect of MIP on T2DM needs further study, and the biological activity of MIP in the digestion process and the actual digestion in vivo also need more thorough research.

## Figures and Tables

**Table 1:** Changes in the molecular weight of MIP during digestion.

Sample	Fraction	Mw (kDa)	Mn (kDa)	Mw/Mn
MIP	1	398.2	388.7	1.024
	2	21.5	20.7	1.035
MIP-S	1	32.3	23.8	1.357
MIP-G	1	19.1	14.9	1.286
	2	11.6	8.5	1.369
MIP-I	1	21.9	16.8	1.304
	2	11.7	8.5	1.369

MIP, *Morchella importuna* polysaccharide; MIP-S, MIP-G and MIP-I represent the MIP of salivary digestion, salivary gastric digestion and salivary gastrointestinal digestion, respectively. The weight-average molecular weight (Mw) is equal to the molecular weight of each component multiplied by the mass fraction of its components in the whole. The number-average molecular weight (Mn) is equal to the molecular weight of each component multiplied by the molar fraction of each component. Mw/Mn is the polydispersity index, which is used to measure the breadth of the polymer molecular weight distribution.

## Data Availability

The original contributions presented in the study are included in the article, further inquiries can be directed to the corresponding author.
